# PAC-ZNN for Robust Target Tracking in WSNs Against Complex Polynomial Noise

**DOI:** 10.3390/s26092774

**Published:** 2026-04-29

**Authors:** Ziying Zhan, Zhiyuan Song, Songjie Huang, Qin Xie, Xiuchun Xiao

**Affiliations:** School of Electronic and Information Engineering, Guangdong Ocean University, Zhanjiang 524088, China; 13537181005@stu.gdou.edu.cn (Z.Z.); songzy384@stu.gdou.edu.cn (Z.S.); hsj@stu.gdou.edu.cn (S.H.); xiaoxc@gdou.edu.cn (X.X.)

**Keywords:** wireless sensor networks, polynomial anti-noise compensation, zeroing neural network, nonlinear activation function

## Abstract

In wireless sensor networks (WSNs), angle of arrival (AOA) and time difference of arrival (TDOA) localization systems relying on distributed sensor measurements degrade significantly under high-order time-varying noise. Although traditional zeroing neural networks (ZNNs) handle dynamic localization tasks, their insufficient robustness against such high-order noise often compromises convergence stability and accuracy. To address this limitation, this paper proposes a polynomial anti-noise compensation ZNN (PAC-ZNN) incorporating a polynomial anti-noise compensation (PAC) term and a logarithmic mapping activation function (LMAF). Specifically, the PAC term mitigates the adverse effects of cumulative high-order noise, while the LMAF further enhances the convergence speed and stability of the system. The global convergence and robustness of the proposed PAC-ZNN are rigorously proven based on Lyapunov stability theory. Simulation results demonstrate that when applied to AOA and TDOA-based dynamic localization tasks, the proposed PAC-ZNN outperforms traditional ZNN-based solutions in terms of anti-noise capability, convergence efficiency, and localization precision under high-order noise conditions. Furthermore, it maintains robust tracking performance even under complex multipath environments, verifying its superior performance and practical application value.

## 1. Introduction

With the widespread adoption of the Internet of Things, wireless sensor networks (WSNs) have become a key technology driving its development [1,2]. In specific deployment environments, WSNs offer significant advantages such as relatively low cost [3], compact size, and high fault tolerance [4]. Multiple sensor nodes perform data sensing, precise processing, and aggregation before transmitting information to the platform [5]. At its core, data fusion reduces redundant interference [6], enhancing data accuracy and fault tolerance [7]. In many well-designed systems, its self-organizing and reliable nature enables remote monitoring and management of physical characteristics within the collection area [8,9].

Target tracking, as one of the core technologies enabling position awareness in WSNs, has found extensive applications across numerous fields, including urban traffic navigation [10], aerospace exploration [11], and wildlife tracking [12]. Early target tracking methods primarily relied on fluctuations in physical quantities like received signal strength and acoustic amplitude variations [13] to infer target positions. However, these approaches could not provide precise coordinate information, resulting in overall low localization accuracy. To enhance system performance, researchers introduced statistical methods such as Kalman filtering into WSNs [14], enabling localization technology to exhibit greater robustness in high-noise environments [15]. This, however, also introduced significant computational complexity and energy consumption overhead. Subsequently, to adapt to complex scenarios involving high noise and multipath propagation, researchers proposed more robust tracking solutions, such as iterative tracking methods based on optimization theory [16] and auxiliary tracking techniques incorporating geometric constraints [17]. However, due to the real-time, continuous, and dynamic nature of target tracking tasks in WSNs, traditional numerical methods have gradually revealed significant limitations in the solution process. Not only do they struggle to meet the real-time tracking requirements [18] of dynamic sensor networks, but they are also prone to introducing tracking errors.

In recent years, neural dynamics [19,20,21] have been widely applied to solving various problems [22,23,24] due to their dynamic online solution capabilities [25,26], parallel distributed computing features [27,28], and strong robustness achieved through function regularization [29,30] and adaptive weight adjustment [31,32,33]. Zeroing neural networks (ZNNs) [34,35,36] leverage derivative information to overcome hysteresis errors [37,38] and enhance solution accuracy. However, constrained by linear first-order evolution equations, they exhibit slow convergence and insufficient noise immunity [39,40]. Addressing this, Xiao et al. proposed a finite-time zeroing neural network (FTZNN) [41,42], which achieves finite-time convergence through a nonlinear dual-driver structure. Nevertheless, residual error convergence remains suboptimal under noisy conditions. Jin et al. designed the noise suppression recurrent neural network (NSRNN) [43], employing saturated permissible activation functions to break traditional constraints. Under constant and bounded random noise, it reduces steady-state error to a low order of magnitude, meeting high-precision control requirements. Subsequent researchers proposed the noise-tolerant zeroing neural network (NTZNN) [44], which incorporates a time-varying derivative compensation mechanism to ensure approximation accuracy during rapid coefficient matrix changes. This enables controllable and optimizable residual error under time-varying linear and bounded random noise. However, while these models effectively solve dynamic localization problems under simple noise scenarios, their accuracy still has room for improvement. In high-order noise scenarios [45], ZNN, FTZNN, and NSRNN all struggle to provide effective disturbance rejection.

**Figure 1:**
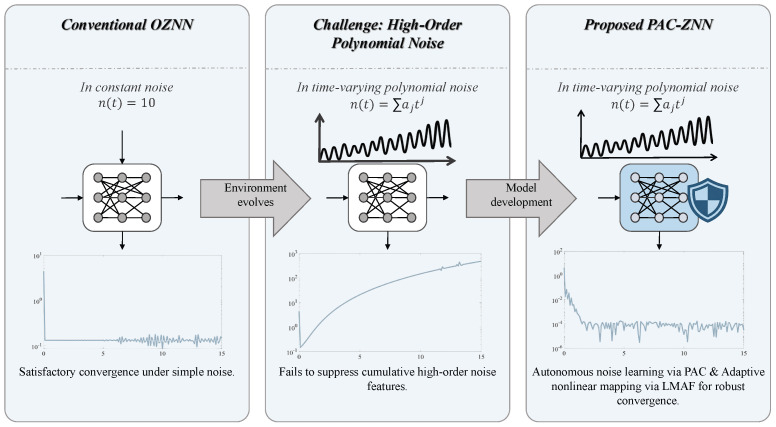
Design motivation of the PAC-ZNN addressing conventional OZNN instability in high-order polynomial noise.

**Figure 2:**
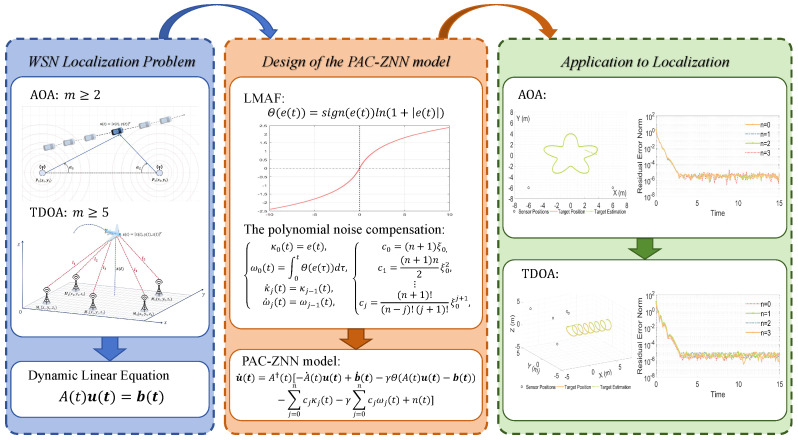
Implementation flowchart of the PAC-ZNN applied to dynamic WSN localization tasks.

To address the aforementioned challenges, this paper proposes a polynomial anti-noise compensation ZNN (PAC-ZNN), which incorporates the polynomial anti-noise compensation (PAC) term [45] for WSN localization using angle of arrival (AOA) and time difference of arrival (TDOA) algorithms. Figure 1 visually illustrates the design motivation and core concepts of PAC-ZNN. The core of this model lies in introducing a PAC term based on the Taylor series expansion principle, which achieves noise cancellation by actively learning noise features. This mechanism significantly reduces the dependence of the model on noise information, markedly enhancing its versatility and practicality. In high-order noise scenarios, the PAC-ZNN demonstrates superior noise resistance compared to existing models, effectively broadening its noise suppression range. Additionally, this paper introduces a nonlinear logarithmic mapping activation function (LMAF). This function prevents model divergence caused by violent error fluctuations during burst noise or severe interference. It also adaptively adjusts convergence speed according to different error magnitudes, achieving precise residual error elimination. Figure 2 systematically illustrates the complete implementation framework of PAC-ZNN for solving dynamic positioning problems. Specifically, compared to ZNN, FTZNN, and NSRNN, the proposed PAC-ZNN demonstrates superior convergence performance and robustness in various unknown high-order complex noise environments. In addition, to better simulate real-world deployment environments, we have extended our performance evaluation to include dynamic localization simulations under multipath effects. Even when simultaneously confronted with measurement errors caused by multipath propagation and complex higher-order noise interference, PAC-ZNN continues to deliver high-precision trajectory tracking and localization performance.

The core contributions of this study can be summarized as follows:

1. We propose a PAC-ZNN for AOA and TDOA-based WSN localization. Integrating a PAC term and LMAF mitigates high-order noise and enhances convergence stability, overcoming the limited robustness of traditional ZNNs.
2. Theoretical analysis proves the global convergence and stability of the PAC-ZNN, ensuring its residual error ultimately converges to zero.
3. Computer simulation experiments validate the superiority and practicality of PAC-ZNN compared to existing models.

Next, we outline the structure of the remaining sections in this paper. Section 2 derives the calculation formulas for AOA and TDOA algorithms and presents design formulas for several common and typical comparison models. Section 3 proposes the PAC-ZNN constructed in this paper. Section 4 theoretically verifies the global convergence and robustness of the PAC-ZNN. Section 5 validates the convergence performance and strong robustness of PAC-ZNN under different orders of noise and multipath environments through simulation experiments.

## 2. Related Work

### 2.1. WSN Localization Problem

In the field of target tracking and localization, researchers have developed various algorithms based on different localization principles, which can be broadly categorized into distance-based, distance-independent [46], and hybrid types. Distance-based localization algorithms estimate target locations by extracting physical signal parameters, with typical examples including TDOA [17,47], AOA [48], and Received Signal Strength [49]. These methods can achieve high localization accuracy, reaching centimeter-level precision in certain scenarios. In contrast, distance-independent algorithms (e.g., DV-Hop [50]) infer target locations using only network connectivity and topological relationships, offering low hardware requirements, reduced deployment cost, and strong interference resistance. Hybrid localization algorithms [51] integrate the strengths of multiple methods to further improve overall localization performance.

Considering the low-cost, low-power, and large-scale deployment characteristics of WSN, localization algorithms should be adapted accordingly. Among them, AOA provides direction information and is commonly applied in two-dimensional scenarios, requiring at least two base stations (m≥2). TDOA provides high accuracy and is suitable for three-dimensional scenarios, determining target coordinates via time-difference measurements and typically requiring at least five base stations (m≥5) to effectively guarantee both solvability and positioning accuracy.

#### 2.1.1. AOA-Based Localization Principle

Referring to [17], we derive the AOA-based localization method. In two-dimensional space, the AOA algorithm measures the azimuth angle $\left(\alpha_{i}\left(t\right)\right)$ of the received signal at each anchor sensor node and estimates the target coordinates (x,y) by exploiting geometric relationships. We define the position vector of the target node and the position matrix of the *m* anchor sensor nodes as follows:

$$u\left(t\right)=\left[\begin{array}{c} x(t)\\ y(t) \end{array}\right]\in R^{2},\;P=\left[\begin{array}{cccc} x_{1} & x_{2} & \ldots & x_{m}\\ y_{1} & y_{2} & \ldots & y_{m} \end{array}\right]\in R^{2\times m}.$$

For the *i*-th anchor, the value of $\alpha_{i}\left(t\right)$ is defined by the horizontal differences $\left(\Delta x_{i},\Delta y_{i}\right)$:

(1) $$\begin{array}{cc} \tan(\alpha_{i}\left(t\right)) & =\frac{\Delta y_{i}}{\Delta x_{i}}=\frac{y\left(t\right)-y_{i}}{x\left(t\right)-x_{i}}, \end{array}$$

where i∈{1,2,⋯,m}, the above Equation (1) can be rearranged in the following form:

(2) $$\begin{array}{c} -\tan\left(\alpha_{i}\left(t\right)\right)x\left(t\right)+y\left(t\right)=-x_{i}\tan\left(\alpha_{i}\left(t\right)\right)+y_{i}. \end{array}$$

According to Equation (2), we can integrate the equations of m anchor sensor nodes into matrix form, expressed as

(3) $$\left[\begin{array}{cc} -\tan\alpha_{1} & 1\\ \vdots & \vdots \\ -\tan\alpha_{m} & 1 \end{array}\right]\left[\begin{array}{c} x(t)\\ y(t) \end{array}\right]=\left[\begin{array}{c} -x_{1}\tan\alpha_{1}+y_{1}\\ \vdots \\ -x_{m}\tan\alpha_{m}+y_{m} \end{array}\right].$$

**Figure 3:**
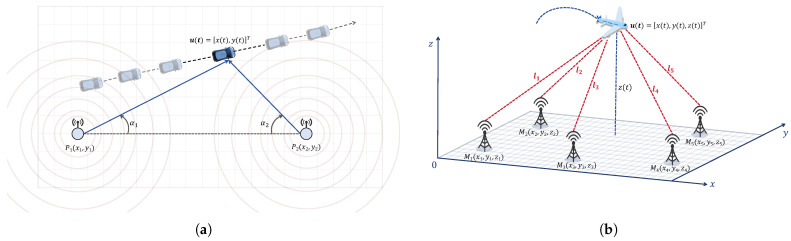
Schematic diagrams of the geometric measurement models for WSN localization: (**a**) two-dimensional localization model based on AOA; (**b**) three-dimensional localization model based on TDOA.

As illustrated in Figure 3a, the relative geometric relationship between the target and anchors defines the mathematical AOA measurement model.

#### 2.1.2. TDOA-Based Localization Principle

Referring to [17], we derive the TDOA-based localization method. In the TDOA algorithm, the position vector of the target node and the position matrix of *m* anchor sensor nodes are defined as

$$u\left(t\right)=\left[\begin{array}{c} x(t)\\ y(t)\\ z(t) \end{array}\right]\in R^{3},\;M=\left[\begin{array}{cccc} x_{1} & x_{2} & \cdots & x_{m}\\ y_{1} & y_{2} & \cdots & y_{m}\\ z_{1} & z_{2} & \cdots & z_{m} \end{array}\right]\in R^{3\times m}.$$

In three-dimensional scenarios, TDOA takes the first anchor as the reference and estimates the target coordinates from the measured arrival-time differences, together with the signal propagation velocity. The squared separation between the target node and the *i*-th anchor sensor node is

(4) $$\begin{array}{c} l_{i}^{2}\left(t\right)=R_{i}-2x_{i}x\left(t\right)-2y_{i}y\left(t\right)-2z_{i}z\left(t\right)+x^{2}\left(t\right)+y^{2}\left(t\right)+z^{2}\left(t\right) \end{array}$$

where $R_{i}=x_{i}^{2}+y_{i}^{2}+z_{i}^{2}$. Similarly, we obtain $l_{1}^{2}\left(t\right)$. Subtracting $l_{1}^{2}\left(t\right)$ from (4) gives

(5) $$l_{i}^{2}\left(t\right)-l_{1}^{2}\left(t\right)=R_{i}-R_{1}-2x_{i1}x\left(t\right)-2y_{i1}y\left(t\right)-2z_{i1}z\left(t\right),$$

with $l_{i1}=l_{i}-l_{1}$, $x_{i1}=x_{i}-x_{1}$, $y_{i1}=y_{i}-y_{1}$, $z_{i1}=z_{i}-z_{1}$, Equation (5) can be rewritten as

(6) $$\begin{array}{cc} l_{i}^{2}\left(t\right)-l_{1}^{2}\left(t\right) & =l_{i1}^{2}\left(t\right)+2l_{1}\left(t\right)l_{i1}\left(t\right). \end{array}$$

Combining Equations (5) and (6) yields

(7) $$x_{i1}x\left(t\right)+y_{i1}y\left(t\right)+z_{i1}z\left(t\right)+2l_{1}\left(t\right)l_{i1}\left(t\right)=\frac{1}{2}\left[R_{i}-R_{1}-l_{i1}^{2}\left(t\right)\right].$$

Stacking the equations for *m* anchors leads to

(8) $$\left[\begin{array}{cccc} x_{21} & y_{21} & z_{21} & l_{21}\\ x_{31} & y_{31} & z_{31} & l_{31}\\ \vdots & \vdots & \vdots & \vdots \\ x_{m1} & y_{m1} & z_{m1} & l_{m1} \end{array}\right]\left[\begin{array}{c} x(t)\\ y(t)\\ z(t)\\ l_{1} \end{array}\right]=\frac{1}{2}\left[\begin{array}{c} R_{2}-R_{1}-l_{21}^{2}\\ R_{3}-R_{1}-l_{31}^{2}\\ \vdots \\ R_{m}-R_{1}-l_{m1}^{2} \end{array}\right]$$

As illustrated in Figure 3b, the geometric relationship between the anchor and target defines the TDOA measurement model.

### 2.2. ZNN-Based WSN Localization Algorithms

Overcoming the limitations of traditional numerical methods such as Gaussian elimination [52] and Jacobian iteration [53], researchers have extensively applied neural dynamics methods across various engineering fields [54]. Models represented by ZNN solve continuous-time dynamic nonlinear problems in real time by constructing error dynamics equations [55]. Their core advantage lies in forcing the target problem to satisfy an exponentially decaying differential equation, ensuring residual errors do not accumulate over time. This enables rapid response in high-frequency scenarios, circumventing the time-lag errors inherent in traditional methods. Building upon this foundation, researchers have iteratively refined models tailored to diverse noise environments and performance requirements, significantly enhancing solution robustness and accuracy. For instance, NAFTCZNN [56] achieves finite-time convergence with an optimal upper bound through an enhanced nonlinear symbolic dual-power activation function. In the WSN domain of target tracking and localization, the MZDL1 and MZDL2 [17] incorporate nonlinear activation functions to effectively resist environmental dynamic disturbances, yet struggle to adapt to high-noise scenarios. In response, the STZNN [57] integrates the super-distortion algorithm with ZNN, demonstrating strong anti-interference capabilities against constant noise and dynamically bounded noise. However, when confronted with complex scenarios like high-order time-varying noise, these models struggle to perform effectively, failing to withstand intense and rapidly fluctuating noise disturbances.

### 2.3. Analysis of Previous Models

#### 2.3.1. OZNN

The OZNN updates the error function iteratively along the gradient-descent direction, enabling efficient handling of time-varying problems. Consequently, the corresponding residual error dynamics are given by

(9) $$\dot{e}\left(t\right)=\frac{de(t)}{dt}=-\gamma e\left(t\right),$$

where γ>0 controls the convergence rate. This evolutionary dynamics design ensures that the residual norm converges globally and exponentially to zero, independent of the initial values of the system. Specifically, larger values of γ accelerate the convergence process, thereby enhancing the model’s real-time solution performance.

#### 2.3.2. FTZNN

The FTZNN solves time-varying problems with finite-time convergence, allowing the state to reach the non-stationary solution within a bounded time and reducing tracking errors caused by convergence delay in fast dynamic localization. The evolution equation is expressed in the following form:

(10) $$\dot{e}\left(t\right)=-\beta_{1}e\left(t\right)-\beta_{2}e^{q/p}\left(t\right),$$

where $\beta_{1}>0$, $\beta_{2}>0$, and p>q. When the error is large, $-\beta_{1}e\left(t\right)$ plays a significant role, ensuring a rapid decay rate. Conversely, when the residual error is small, $-\beta_{2}e^{q/p}\left(t\right)$ dominates, significantly accelerating the convergence process and thereby achieving finite-time convergence performance.

#### 2.3.3. NTZNN

Unlike the OZNN, which uses only negative gradient terms to drive residual convergence, the NTZNN introduces an integral term to accumulate historical errors, thereby suppressing noise and improving the robustness of the system. The NTZNN evolves as follows:

(11) $$\dot{e}\left(t\right)=-\gamma e\left(t\right)-\lambda \int_{0}^{t}e\left(u\right)\;du,$$

where γ>0 follows the same positivity condition as in previous models. The term $-\lambda \int_{0}^{t}e\left(u\right)\;du$ performs time integration of the error, generating a compensation signal to counteract persistent external noise. This specific mechanism endows the model with superior noise suppression capabilities.

## 3. Methodology

### 3.1. Preliminaries and Formulation

We uniformly rewrite Equations (3) and (8), ultimately derived from the AOA and TDOA algorithms in the wireless sensor network localization problem, into a dynamic linear form for easier solution. Its expression is

(12) A(t)u(t)=b(t).

Accordingly, the residual error function is defined as

(13) e(t)=A(t)u(t)−b(t).

The objective of (13) is to ensure that the target position vector u(t) can asymptotically approach its theoretical value $u^{*}\left(t\right)$ within a given time interval. According to this requirement, the residual error function e(t) is expected to progressively approach zero. To proceed with the theoretical design and analysis of the proposed PAC-ZNN, the following fundamental assumptions regarding the dynamic linear Equation (12) must be established:

**Assumption 1.**  *The geometric matrix A(t) and measurement vector b(t) are continuously differentiable with respect to time t*.

**Assumption 2.**  *The geometric matrix A(t) maintains full column rank at any time t to ensure the existence and computability of its pseudoinverse $A^{†}\left(t\right)$*.

### 3.2. Design of the PAC-ZNN

In practical application environments, complex and variable noise disturbances are ubiquitous, and such interference can severely impact the localization accuracy of wireless sensor networks. Traditional localization neuro-dynamical models often struggle to effectively suppress the negative effects of high-order time-varying noise. For instance, thermal noise causes slow drift in the gain of receiving sensors, leading to a near-third-order temperature drift error. Signal multipath effects generate nonlinear delay errors during propagation, leading to the accumulation of noise in a high-order polynomial form. To address these challenges, this paper proposes a PAC-ZNN based on a logarithmic mapping activation function. Applied to wireless sensor network localization systems, this model employs a PAC term to effectively suppress complex noise interference, significantly enhancing localization stability as well as accuracy.

#### 3.2.1. Construct LMAF Structure and Form

Reference [17] introduces a nonlinear double-exponential activation function and a double-power activation function. While these functions enhance the dynamic robustness of the model and improve localization accuracy, they may still face challenges in effectively handling noisy interference scenarios. In particular, these functions may struggle with precise control over the convergence direction, especially under large residuals, which could lead to slower convergence or even divergence. In order to address these limitations, we propose a nonlinear LMAF, which produces a gain effect specifically designed for noisy interference conditions. The expression of the LMAF is as follows:

(14) Θ(e(t))=sign(e(t))ln(1+|e(t)|)

This mechanism functions in the following manner:

The LMAF employs a piecewise design to precisely preserve the sign information of residual errors, effectively preventing divergence between the iteration direction and convergence direction. Its segmented structure incorporates the sign(e(t)) mechanism, ensuring error feedback consistently acts along the residual decay direction to guarantee successful convergence targeting.

Simultaneously, the introduction of logarithmic terms enables nonlinear mapping of errors, establishing an adaptive modulation mechanism based on |e(t)|. This dynamically adjusts feedback strength according to residual states. This design significantly mitigates steep gradient growth under large residuals while preserving gradient sensitivity for small residuals, thereby achieving rapid convergence and enhancing steady-state accuracy.

#### 3.2.2. Incorporating the PAC Term

In practical application scenarios, noise characteristics exhibit unpredictability and significant time-varying properties, posing a severe challenge to traditional noise reduction methods. Most existing models rely on predefined noise patterns, yet such models struggle to adapt to dynamically changing interference in real-time scenarios. To address this issue, we introduce a PAC term, expressed as follows:

(15) $$\begin{array}{cc} \left\{\begin{array}{c} \kappa_{0}\left(t\right)=e\left(t\right),\\ \omega_{0}\left(t\right)=\int_{0}^{t}\Theta \left(e\left(\tau \right)\right)d\tau ,\\ {\dot{\kappa }}_{j}\left(t\right)=\kappa_{j-1}\left(t\right),\\ {\dot{\omega }}_{j}\left(t\right)=\omega_{j-1}\left(t\right), \end{array}\right.\; & \left\{\begin{array}{c} c_{0}=\left(n+1\right)\xi_{0},\\ c_{1}=\frac{(n+1)n}{2}\xi_{0}^{2},\\ \vdots \\ c_{j}=\frac{(n+1)!}{(n-j)!(j+1)!}\xi_{0}^{j+1}, \end{array}\right. \end{array}$$

where j∈{1,2,⋯,n}. This PAC achieves highly effective interference suppression through precise fitting to the noise function. Utilizing iterative calculations based on residual error function and LMAF, it not only accurately eliminates interference but also ensures the algorithm possesses dynamic real-time solving capabilities.

By jointly utilizing the mathematical relations in Equations (13)–(15), the resulting formulation of the PAC-ZNN can be systematically derived, and its explicit dynamic expression is presented as follows:

(16) $$\begin{array}{c} \dot{u}\left(t\right)=A^{†}\left(t\right)\left[-\dot{A}\left(t\right)u\left(t\right)+\dot{b}\left(t\right)-\gamma \Theta \left(A\left(t\right)u\left(t\right)-b\left(t\right)\right)-\sum_{j=0}^{n}c_{j}\kappa_{j}\left(t\right)-\gamma \sum_{j=0}^{n}c_{j}\omega_{j}\left(t\right)+\eta \left(t\right)\right]. \end{array}$$

This formulation combines LMAF and PAC to ensure robust global convergence.

### 3.3. System Implementation

In the simulation implementation phase, this paper converts the constructed PAC-ZNN continuous-time dynamic equation into a first-order differential form and employs the ode45 solver in MATLAB R2024a to perform the integration (see Algorithm 1 for details). The specific implementation process is as follows: First, the target position components, instantaneous residual error, and variables of the PAC term are integrated into a unified state vector, while the integration interval t∈[0,15] is set as the solution range for ode45. Subsequently, a system function is constructed to ensure that at each time step, the geometric matrix A(t), vector b(t), and their derivatives can be computed based on the current time. This enables the update of the residual error $e\left(t\right)=A\left(t\right)u_{\mathrm{pos}}-b\left(t\right)$, the nonlinear activation function, and the PAC-ZNN dynamic equation incorporating the polynomial anti-noise compensation term. Ultimately, all state components can be defined consistently through $\dot{x}=f\left(t,x\right)$.
**Algorithm 1** Pseudo-code of the ode45-based PAC-ZNN implementation for dynamic TDOA localization**Require:** Functions $A\left(t\right),b\left(t\right),\dot{A}\left(t\right),\dot{b}\left(t\right)$; Parameters $\gamma =5,n=4,\xi_{0}=10$; Noise η(t)  1:**Initialize** $x\left(0\right)=[u_{\mathrm{pos}}\left(0\right);\;e\left(0\right);\;q_{1}\left(0\right);\ldots ;q_{9}\left(0\right)]$, where q denotes the coefficients in Equation (15).  2:**Integrate** the system using ode45: $\dot{x}\left(t\right)=f\left(t,x\right)$, where the function f(t,x) is defined as steps 3–10:  3:**Compute** $A\left(t\right),b\left(t\right),\dot{A}\left(t\right),\dot{b}\left(t\right)$.  4:**Extract** $u_{pos},q_{1},\ldots ,q_{9}$ from current state vector x.  5:**Compute residual** $e\left(t\right)=A\left(t\right)u_{pos}\left(t\right)-b\left(t\right)$.  6:**Compute activation** Θ(e(t))=sign(e(t))ln(1+|e(t)|), as in Equation (14).  7:**Compute coefficients** $\left\{c_{0},\ldots ,c_{4}\right\}$ by Equation (15).  8:**Compute** ${\dot{u}}_{pos}\left(t\right)$ according to Equation (16):$${\dot{u}}_{pos}\left(t\right)=A^{†}\left(t\right)\left[-\dot{A}\left(t\right)u_{pos}\left(t\right)+\dot{b}\left(t\right)-\gamma \Theta \left(e\left(t\right)\right)-\sum_{j=0}^{4}c_{j}\left(q_{j}+\gamma q_{j+5}\right)+\eta \left(t\right)\right]$$  9:**Update compensation states**:       ${\dot{q}}_{1}=e\left(t\right),\;{\dot{q}}_{2}=q_{1},\;{\dot{q}}_{3}=q_{2},\;{\dot{q}}_{4}=q_{3},$       ${\dot{q}}_{5}=\Theta \left(e\left(t\right)\right),\;{\dot{q}}_{6}=q_{5},\;{\dot{q}}_{7}=q_{6},\;{\dot{q}}_{8}=q_{7},\;{\dot{q}}_{9}=q_{8}$.10:**Return** derivative vector $\dot{x}\left(t\right)=\left[{\dot{u}}_{pos}\left(t\right);\;e\left(t\right);\;{\dot{q}}_{1}\left(t\right);\ldots ;{\dot{q}}_{9}\left(t\right)\right]$.

### 3.4. Comparison Among Existing Neural Network Models for Localization

This section compares several existing localization neural network models and uniformly defines the error function for dynamic localization as e(t). First, the traditional lagrange programming neural network (LPNN) [58] updates along the negative gradient of the objective function, expressed as

(17) $$\dot{u}\left(t\right)=-\gamma \nabla L\left(u\left(t\right)\right),$$

where ∇L(u(t)) denotes the gradient of the Lagrangian function. To mitigate dynamic lag errors, the ZNN method is introduced, utilizing time derivatives to ensure error decay. To enhance convergence speed and robustness against interference in dynamic positioning, the modified zeroing neurodynamics (MZND) [17] incorporates two nonlinear activation functions. Its evolution process is defined as follows:

(18) $$\dot{e}\left(t\right)=-\gamma \Phi_{1,2}\left(e\left(t\right)\right).$$

Here, $\Phi_{1}\left(e\left(t\right)\right)=\kappa_{1}{\left|e\left(t\right)\right|}^{\tau }\mathrm{sgn}\left(e\left(t\right)\right)+\kappa_{2}{\left|e\left(t\right)\right|}^{\sigma }\mathrm{sgn}\left(e\left(t\right)\right)$ corresponds to the activation function of MZDL1, while $\Phi_{2}\left(e\left(t\right)\right)=\rho \left(\exp\left(\theta e\left(t\right)\right)-\exp\left(-\theta e\left(t\right)\right)\right)$ corresponds to the activation function of MZDL2. To converge the error to zero within a finite time, the finite-time reciprocal zeroing neural network (FTRZNN) [59] employs a fractional power proportional feedback design. It defines an energy function $\Phi \left(t\right)=\frac{1}{2}{∥e\left(t\right)∥}_{2}^{2}$ and evolves it to satisfy

(19) $$\dot{\Phi }\left(t\right)=-\frac{\gamma }{2}{∥e\left(t\right)∥}_{2}^{\alpha }.$$

To address constant and low-order linear noise in real-world environments, noise-immune zeroing neural dynamics (NIZND) [60] incorporates a passive cumulative error integration term term defined as follows:

(20) $$\dot{e}\left(t\right)=-\gamma_{1}e\left(t\right)-\gamma_{2}\int_{0}^{t}e\left(\tau \right)d\tau .$$

In contrast, the noise-suppressing recurrent neural network (NSRNN) [43] combines an integral structure with a saturated permissible activation function to limit the output amplitude, as expressed by the formula:

(21) $$\dot{e}\left(t\right)=-\nu F_{\varphi_{1}}\left(e\left(t\right)\right)-\mu F_{\varphi_{2}}\left(e\left(t\right)+\nu \int_{0}^{t}F_{\varphi_{1}}\left(e\left(\tau \right)\right)d\tau \right).$$

For illustrating the advantages of the proposed model, a detailed characteristic comparison between PAC-ZNN and other neurodynamics models is summarized in Table 1.

## 4. Theoretical Analysis

In the preceding sections, we constructed the PAC-ZNN (16) to address residual errors from high-order noise and unknown coefficients in dynamic localization. This section will rigorously analyze and derive the model’s convergence and robustness through theoretical proof, providing reliable engineering validation for its practical application. This theoretical demonstration simultaneously confirms the PAC-ZNN’s (16) effectiveness and reliability in solving wireless sensor network localization problems within subsequent external high-order environments. Note that these proofs rely on the assumptions from Section 3.1, specifically the smoothness and full column rank of A(t). To facilitate the reading of the theoretical proof that follows, the main symbols used in this section are summarized in Table 2.

**Theorem 1.**  *For the PAC-ZNN (16), the characteristic equation is ${\left(s+\xi_{0}\right)}^{n+1}$ with $\xi_{0}>0$. Since all its roots lie in the left-half plane of the complex plane, the system is asymptotically stable. Therefore, the PAC-ZNN is robust*.

**Proof of Theorem 1.**  When polynomial noise $\left(\eta \left(t\right)=\sum_{j=0}^{r}z_{j}\left(t\right)=\sum_{j=0}^{r}a_{j}t^{j}\right)$ interference is present, the expression for the PAC-ZNN can be written as

(22) $$\dot{e}\left(t\right)=-\gamma \Theta \left(e\left(t\right)\right)-\sum_{j=0}^{n}c_{j}\kappa_{j}\left(t\right)-\gamma \sum_{j=0}^{n}c_{j}\omega_{j}\left(t\right)+\sum_{j=0}^{r}a_{j}t^{j}.$$

We define an intermediate state variable:

(23) $$\rho_{j}\left(t\right)=\kappa_{j}\left(t\right)+\gamma \omega_{j}\left(t\right),\;\forall j=0,1,2,\ldots ,n.$$

By making use of the recurrence relationship established in (15), the corresponding derivative form of Equation (23) can be further deduced:

(24a) $$\begin{array}{cc} {\dot{\rho }}_{j}\left(t\right) & =\kappa_{j-1}\left(t\right)+\gamma \omega_{j-1}\left(t\right)=\rho_{j-1}\left(t\right), \end{array}$$

(24b) $$\begin{array}{cc} L\{{\dot{\rho }}_{j}\left(t\right)\} & =sP_{j}\left(s\right)-\rho_{j}\left(0\right)=P_{j-1}\left(s\right), \end{array}$$

where L{·} denotes the Laplace transform operator. The relationship in (24b) leads to $P_{j}\left(s\right)=\frac{P_{j-1}\left(s\right)+\rho_{j}\left(0\right)}{s}$. We adopt the recurrence method to expand the formula: when j=1, $P_{1}\left(s\right)=\frac{P_{0}\left(s\right)}{s}+\frac{\rho_{1}\left(0\right)}{s}$ is derived from the recurrence relation; when j=2, substituting the result of $P_{1}\left(s\right)$ into the recurrence formula gives $P_{2}\left(s\right)=\frac{P_{0}\left(s\right)}{s^{2}}+\frac{\rho_{1}\left(0\right)}{s^{2}}+\frac{\rho_{2}\left(0\right)}{s}$; when j=3, similarly substituting the result of $P_{2}\left(s\right)$ into the recurrence formula yields $P_{3}\left(s\right)=\frac{P_{0}\left(s\right)}{s^{3}}+\frac{\rho_{1}\left(0\right)}{s^{3}}+\frac{\rho_{2}\left(0\right)}{s^{2}}+\frac{\rho_{3}\left(0\right)}{s}$; repeating the above recurrence process j times, the general formula can be obtained:

(25) $$P_{j}\left(s\right)=\frac{P_{0}\left(s\right)}{s^{j}}+\frac{\rho_{j}\left(0\right)}{s}+\frac{\rho_{j-1}\left(0\right)}{s^{2}}+\cdots +\frac{\rho_{1}\left(0\right)}{s^{j}}.$$

According to Equations (22) and (23), the state variable when j=0 can then be written as

(26a) $$\begin{array}{cc} {\dot{\rho }}_{0}\left(t\right) & =\dot{e}\left(t\right)+\gamma \Theta \left(e\left(t\right)\right)=-\sum_{j=0}^{n}c_{j}\kappa_{j}\left(t\right)-\gamma \sum_{j=0}^{n}c_{j}\omega_{j}\left(t\right)+\sum_{j=0}^{r}z_{j}\left(t\right), \end{array}$$

(26b) $$\begin{array}{cc} L\{{\dot{\rho }}_{0}\left(t\right)\} & =sP_{0}\left(s\right)-\rho_{0}\left(0\right)=-\sum_{j=0}^{n}c_{j}P_{j}\left(s\right)+\sum_{j=0}^{r}Z_{j}\left(s\right), \end{array}$$

where $Z_{j}\left(s\right)$ is the Laplace transform of the polynomial noise component $z_{j}\left(t\right)$. Associate (25) with (26b) and organize $P_{0}\left(s\right)$ to the left side of the equation:

(27) $$sP_{0}\left(s\right)+c_{0}P_{0}\left(s\right)+\sum_{j=1}^{n}c_{j}\frac{P_{0}\left(s\right)}{s^{j}}=\rho_{0}\left(0\right)-\sum_{j=0}^{n}c_{j}\left(\frac{\rho_{1}\left(0\right)}{s^{j}}+\cdots +\frac{\rho_{j}\left(0\right)}{s}\right)+\sum_{j=0}^{r}Z_{j}\left(s\right).$$

We define $D\left(s\right)=s^{n+1}+c_{0}s^{n}+c_{1}s^{n-1}+\cdots +c_{n}$ and multiply both sides of Equation (27) by $s^{n}$ simultaneously to obtain

(28) $$P_{0}\left(s\right)=\frac{1}{D(s)}[s^{n}\rho_{0}\left(0\right)-s^{n-1}\left(c_{1}\rho_{1}\left(0\right)+\cdots +c_{n}\rho_{n}\left(0\right)\right)-\cdots -c_{n}\rho_{1}\left(0\right)]+\frac{1}{D(s)}s^{n}\sum_{j=0}^{r}Z_{j}\left(s\right).$$

Let $y\left(t\right)={\dot{\rho }}_{0}\left(t\right)$ be the total system response. The Laplace transform is formulated as

(29) $$\begin{array}{cc} Y(s) & =sP_{0}\left(s\right)-\rho_{0}\left(0\right)\\ \; & =\frac{1}{D(s)}[s(s^{n}\rho_{0}\left(0\right)-s^{n-1}\sum_{i=1}^{n}c_{i}\rho_{i}\left(0\right)\\ \; & \;\;-\cdots -c_{n}\rho_{1}\left(0\right))-\rho_{0}\left(0\right)D\left(s\right)]\\ \; & \;+\frac{s^{n+1}}{D(s)}\sum_{j=0}^{r}Z_{j}\left(s\right). \end{array}$$

In the system response expressed by Equation (29), the first term is interpreted as the zero-input component, whereas the second term represents the zero-state contribution. Accordingly, the transfer function of the system is characterized as the quotient between the Laplace transform of the zero-state response and that of the input noise. On this basis, the following relationship is derived:

$$H\left(s\right)=\frac{\left(s^{n+1}\sum_{j=0}^{r}Z_{j}\left(s\right)\right)/D\left(s\right)}{\sum_{j=0}^{r}Z_{j}\left(s\right)}=\frac{s^{n+1}}{s^{n+1}+c_{0}s^{n}+c_{1}s^{n-1}+\cdots +c_{n}}.$$

We define $s^{n+1}+c_{0}s^{n}+c_{1}s^{n-1}+\cdots +c_{n}={\left(s+\xi_{0}\right)}^{n+1}=\sum_{j=0}^{n+1}\frac{(n+1)!}{j!(n+1-j)!}\xi_{0}^{n+1-j}s^{j}$. When $\xi_{0}>0$ is taken as a constant, all poles of the transfer function H(s) are ensured to lie in the left-half complex plane. This pole placement immediately establishes the system’s stability, as such a configuration guarantees that every mode of the system decays over time. □

**Remark 1.**  *A larger $\xi_{0}$ shifts the poles farther from the imaginary axis, theoretically accelerating the exponential decay of the zero-input response and thereby achieving faster transient response. However, from a robust control perspective, an excessively large $\xi_{0}$ may cause the system to fall into severe overdamping conditions and amplify its sensitivity to high-frequency disturbances. Increasing γ enhances the driving force toward the theoretical solution, but an overly aggressive γ may induce chattering effects during implementation. In Section 5.3, we conducted a detailed parameter sensitivity experiment specifically designed to determine the optimal values of γ and $\xi_{0}$ that minimize the average steady-state residual error*.

**Corollary 1.**  ***(1):**** When n≥r, the characteristic equation $D\left(s\right)={\left(s+\xi_{0}\right)}^{n+1}=0$ always satisfies the strict Hurwitz condition. Therefore, the system converges, i.e., $\lim_{t\to \infty }e\left(t\right)=0$*.

***(2):***  *When the actual noise order r is higher than the compensation order n, the noise can be decomposed into a compensated portion and an uncompensated residual portion:*
$$\eta^{*}\left(t\right)=\sum_{j=0}^{n}a_{j}t^{j}+\Delta \eta \left(t\right),$$
  *where $\Delta \eta \left(t\right)=\sum_{j=n+1}^{r}a_{j}t^{j}$. In practical dynamic localization scenarios, since the target tracking process typically takes place within a finite time window, the noise energy is finite. Therefore, the disturbance term Δη(t) satisfies the boundedness condition, which is expressed as*
sup|Δη(t)|≤ℏ,
  *where ℏ is an unknown positive constant. Introducing Δη(t) into the error equation, the dynamic evolution of the overall system can be expressed as follows:*
$$\dot{e}\left(t\right)=-\gamma \Theta \left(e\left(t\right)\right)+\Delta \eta \left(t\right).$$
  *For the i-th element of the residual error vector $e_{i}\left(t\right)$, we construct the Lyapunov candidate function $V_{i}\left(t\right)=\frac{1}{2}e_{i}^{2}\left(t\right)$, whose time derivative is given by*
$${\dot{V}}_{i}\left(t\right)=e_{i}\left(t\right){\dot{e}}_{i}\left(t\right)=e_{i}\left(t\right)\left[-\gamma \mathrm{sign}\left(e_{i}\left(t\right)\right)\ln(1+|e_{i}\left(t\right)\left|\right)+\Delta \eta_{i}\left(t\right)\right].$$
  *By utilizing the property $e_{i}\left(t\right)\mathrm{sign}\left(e_{i}\left(t\right)\right)=\left|e_{i}\left(t\right)\right|$, we can derive the following form of the mathematical inequality:*
$$\begin{array}{cc} \dot{V}\left(t\right) & \leq -\gamma |e_{i}\left(t\right)|\ln(1+|e_{i}\left(t\right)|)+|e_{i}\left(t\right)||\Delta \eta \left(t\right)|\\ \dot{V}\left(t\right) & \leq -|e_{i}\left(t\right)|\left[\gamma \ln(1+|e_{i}\left(t\right)|)-\hbar \right] \end{array}$$
  *According to Lyapunov stability theory, to ensure that $\dot{V}\left(t\right)<0$, the following condition must be satisfied*
$$\gamma \ln(1+|e_{i}\left(t\right)|)>\hbar .$$
  *This yields the error $|e_{i}\left(t\right)|>\exp\left(\frac{\hbar }{\gamma }\right)-1$. According to the derivation, once the error magnitude $|e_{i}\left(t\right)|$ exceeds the threshold exp(ℏ/γ)−1, $\dot{V}\left(t\right)$ becomes strictly negative-definite. This indicates that the energy of the error system is in a decaying state, which in turn drives the residual $|e_{i}\left(t\right)|$ to decrease. Therefore, the error signal will not diverge to infinity, and the system satisfies ultimate bounded convergence, with the following upper bound:*
$$\underset{t\to \infty }{lim sup}\left|e_{i}\left(t\right)\right|\leq \exp\left(\frac{\hbar }{\gamma }\right)-1.$$

**Theorem 2.**  *On the basis of the proven system stability, employing the final value theorem shows that the residual error of the PAC-ZNN (16) approaches zero, which confirms the convergence of the proposed model*.

**Proof of Theorem 2.**  By invoking the final value theorem, the following relation holds:

(30) $$\begin{array}{cc} \lim_{t\to \infty }y\left(t\right) & =\lim_{s\to 0}sY\left(s\right)\\ \; & =\lim_{s\to 0}\frac{1}{s^{n+1}+c_{0}s^{n}+c_{1}s^{n-1}+\cdots +c_{n}}[\\ \; & \;s^{2}(s^{n}\rho_{0}\left(0\right)-s^{n-1}\left(c_{1}\rho_{1}\left(0\right)+\cdots +c_{n}\rho_{n}\left(0\right)\right)\\ \; & \;-\cdots -c_{n}\rho_{1}\left(0\right))-\rho_{0}\left(0\right)D\left(s\right)]+\lim_{s\to 0}\frac{1}{D(s)}\sum_{j=0}^{r}a_{j}\frac{s^{n}j!}{s^{j+1}}\\ \; & =\lim_{s\to 0}\frac{1}{D(s)}\sum_{j=0}^{r}a_{j}j!s^{n-j+1}=0. \end{array}$$

From the derivation result of (30), it can be explicitly concluded that $y\left(t\right)={\dot{\rho }}_{0}\left(t\right)=\dot{e}\left(t\right)+\gamma \dot{\Theta }\left(e\left(t\right)\right)=0$ holds. This result further verifies that the PAC-ZNN (16), in the presence of noise interference, can still guarantee that the system state starts from arbitrary initial conditions and eventually achieves global convergence to zero. □

**Theorem 3.**  *The computational complexity of the PAC-ZNN model at each iteration step is $O(md^{2}+d^{3}+nm)$. Given that both the spatial dimension d and the compensation order n are very small constants, this complexity can be further simplified to O(m). This indicates that the computational overhead of the algorithm is linearly proportional to the number of sensor anchor nodes*.

**Proof of Theorem 3.**  The computational overhead of the PAC-ZNN is primarily determined by the calculation of the dynamic Equation (16). Let *m* denote the number of anchor sensor nodes, *d* denote the spatial dimension, where d=2 for AOA and d=3 for TDOA, and *n* denote the order of the polynomial noise compensation. The matrices and vectors are dimensioned as $A\left(t\right)\in R^{m\times d}$, $u\left(t\right)\in R^{d\times 1}$, and $b\left(t\right),e\left(t\right)\in R^{m\times 1}$. We analyze the floating point operations, or FLOPs, for each main component as described below.

1. Regarding the residual and derivative terms, the matrix-vector multiplication operations A(t)u(t) and $\dot{A}\left(t\right)u\left(t\right)$ each require 2md−m FLOPs. The subsequent vector addition and subtraction operations used to compute the residual e(t) require O(m) operations.
2. For a nonlinear activation function Θ(e(t)), applying LMAF to an *m*-dimensional vector requires performing sign determination, absolute value calculation, and logarithmic mapping on each element of the vector individually. Assuming the computational cost of these basic nonlinear operations is a constant *f*, the total computational cost of this step is O(m) FLOPs.
3. For the PAC term, computing $\sum_{j=0}^{n}c_{j}\kappa_{j}\left(t\right)$ and $\gamma \sum_{j=0}^{n}c_{j}\omega_{j}\left(t\right)$ involves scalar-vector multiplication and vector addition. For a compensation term of order *n*, this requires approximately 2(2n+1)m FLOPs, with an asymptotic complexity of O(nm).
4. Regarding pseudo-inverse matrix multiplication, computing the pseudo-inverse $A^{†}\left(t\right)={\left(A^{T}\left(t\right)A\left(t\right)\right)}^{-1}A^{T}\left(t\right)$ primarily involves three steps. First, computing the Gram matrix $A^{T}\left(t\right)A\left(t\right)$ requires $O(md^{2})$ floating-point operations. Second, finding the inverse of a d×d matrix requires $O(d^{3})$ floating-point operations. Finally, multiplying $A^{†}\left(t\right)$ by the resulting column vector requires O(md) floating-point operations.

Adding these parts together, the total computational complexity for each iteration step is expressed as

$$O(md^{2}+d^{3}+nm).$$

In practical WSN target tracking scenarios, the spatial dimension is strictly limited to d≤3, while the order of compensation *n* is typically a small integer. Therefore, *d* and *n* can be treated as negligible constants, thereby simplifying the overall computational complexity to O(m). This scaling relationship, which is linear with respect to the number of anchor nodes, confirms the computational efficiency and real-time feasibility of the PAC ZNN model in dynamic localization tasks. □

## 5. Simulation Experiment

Simulations were performed on a computer equipped with an Intel Core i5-13500H (2.60 GHz), 16 GB RAM, Iris Xe Graphics, and Windows 11. The MATLAB R2024a platform was used to conduct the experiments, and the continuous-time ZNN model was numerically solved by utilizing the platform’s built-in “ode45” numerical solver.

### 5.1. Numerical Simulation

It is worth noting that the core of the numerical simulations in this section lies in evaluating the dynamic convergence properties of the model from a mathematical perspective by solving theoretical time-varying linear equations. This fundamental theoretical validation differs fundamentally from the practical simulations for physical localization scenarios in sensor networks discussed later, and it lays a solid theoretical foundation for the latter.

To establish a rigorous performance benchmark, the parameters of all models in the simulation experiments were uniformly set to their respective optimal values. Based on the experimental analysis of parameter sensitivity in Section 5.3, the PAC-ZNN (16) proposed in this paper adopts the optimal design parameters of γ=5 and $\xi_{0}=10$. Additionally, to ensure that the comparison models achieve maximum noise reduction performance, their parameters were configured according to the recommendations in the relevant references. Specifically, based on References [41,42], the parameters for the FTZNN are set to $\beta_{1}=1,\beta_{2}=1$, and q/p=0.2. Furthermore, following the guidance in References [43,44], the parameters for the NTZNN are set to γ=100 and λ=100, while those for the NSRNN are set to μ=10 and v=10. We additionally introduce two representative recent ZNN variants as comparison models. These are the modified gradient RNN (MGRNN) [61] and the nonlinear noise-resistant ZNN (NNR-ZNN) [62]. Following the experimental setup in reference [61], the parameters of the MGRNN were set to α=50 and ϕ=1. Meanwhile, following the simulation conditions in reference [62], the parameters of the NNR-ZNN were set to $\omega_{1}=1$, $\omega_{2}=1$, $k_{1}=1$, $k_{2}=1$, q=0.5, and p=1. It is worth noting that although the optimal parameter values required by these comparison models are typically significantly higher than those of PAC-ZNN (16)—aiming to accelerate convergence and improve accuracy through larger gain coefficients—this inevitably increases computational overhead. In contrast, PAC-ZNN (16) achieves superior robust tracking performance with smaller parameter configurations, fully demonstrating its efficiency in algorithmic design.

In the preceding sections, we have systematically introduced the theoretical principles of the PAC-ZNN (16) and rigorously demonstrated its convergence and robustness from a theoretical perspective. In this section, we conducted comparative analyses with other models through numerical simulation experiments. Together with the clear and comprehensive experimental evidence, the findings demonstrate that the PAC-ZNN (16) performs robustly and achieves superior effectiveness in target-seeking tasks. In the numerical simulation experiments, the dynamic linear Equation (13) employed take the following specific mathematical form:

$$\begin{array}{c} A\left(t\right)=\left[\begin{array}{cccc} \exp(1+t) & \cos(t) & \exp(-t)+\exp(t) & 2+\sin(t)\\ \cos(t) & \exp(1+t) & \exp(-t)+\exp(t) & 2+\sin(t)\\ \exp(-t)+\exp(t) & 2+\sin(t) & \cos(t) & \exp(1+t)\\ 2+\sin(t) & \exp(1+t) & \exp(-t)+\exp(t) & \cos(t) \end{array}\right],\\ b\left(t\right)=\left[\begin{array}{c} 2\cos(t)+\sin(t)\\ 1+t\\ -\sin(t)+t\\ \exp(1-t) \end{array}\right]. \end{array}$$

**Figure 4:**
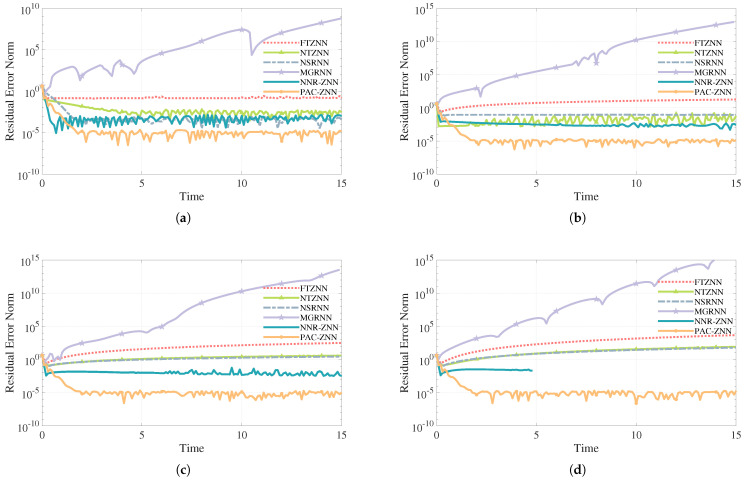
Comparison of different models for solving dynamic linear equation problems in noise environments of different orders: (**a**) In the environment of η(t)=10; (**b**) In the environment of η(t)=10+10t; (**c**) In the environment of $\eta \left(t\right)=10+10t+10t^{2}$; (**d**) In the environment of $\eta \left(t\right)=10+10t+10t^{2}+10t^{3}$.

Figure 4a–d correspond to noise orders progressively increasing from 0 to 3.

#### 5.1.1. Constant Noise Condition

As shown in Figure 4a, PAC-ZNN (16), NSRNN, and NTZNN (11) all exhibit good convergence characteristics under constant noise conditions. Among them, PAC-ZNN (16) achieves the highest convergence accuracy, with residual error stably maintained at the magnitude of $10^{-6}$ to $10^{-5}$. NSRNN follows with residual error stabilizing near $10^{-5}$, while NTZNN’s (11) error fluctuates primarily at the magnitude of $10^{-4}$. In contrast, FTZNN (10) consistently maintained a higher residual level and demonstrated inferior convergence compared to other models. Additionally, NNR-ZNN demonstrates rapid convergence, with residuals successfully reaching $10^{-3}$. In contrast, MGRNN exhibits severe bias, with residuals rapidly diverging to the order of $10^{9}$. This severe divergence is primarily due to the fact that MGRNN is fundamentally derived from gradient evolution formulas and lacks an integration compensation mechanism. Its adaptive gain inadvertently amplifies uncompensated tracking errors, leading to severe numerical divergence. Overall, these comparisons indicate that PAC-ZNN (16) achieves optimal steady-state accuracy under constant noise conditions.

#### 5.1.2. First-Order Noise Condition

Under the first-order noise condition, the PAC-ZNN (16), the NTZNN (11), and the NNR-ZNN demonstrate favorable convergence properties. Notably, in Figure 4b, the NTZNN (11) converges to a stable value within 1 s. Nevertheless, even with increased parameter values of γ and λ, the NTZNN (11) still struggles to attain the high convergence precision and superior stability comparable to the PAC-ZNN (16). The PAC-ZNN (16) maintains a low convergence accuracy of approximately $10^{-5}$ when γ is set to five, with only minor oscillations occurring thereafter. The NTZNN (11) exhibits weaker stability, generating high-frequency fluctuations under noise influence and ultimately achieving inferior convergence precision compared to the PAC-ZNN (16). The FTZNN (10) displays smooth curves with minimal fluctuations but fails to demonstrate effective exponential convergence, unable to reduce residual error. The NSRNN, however, shows a long-term increase in residual error as time-varying noise intensifies, ultimately diverging. In this scenario, the NNR-ZNN demonstrated commendable robustness, with its residuals stabilizing at the $10^{-2}$ level. Meanwhile, the MGRNN continued to diverge, and the extent of its divergence increased as the noise order increased.

#### 5.1.3. Second-Order Noise Condition

As shown in Figure 4c, under second-order noise condition, the NTZNN (11) exhibits divergence driven by noise and can no longer effectively approximate the theoretical solution. Compared to first-order noise interference, the FTZNN (10) and NSRNN exhibit a further overall increase in residual error. The NNR-ZNN still manages to stabilize its residual error at the magnitude of $10^{-2}$ but it exhibits noticeably stronger oscillations than in the first order noise condition. The MGRNN shows a continuous worsening in its divergence magnitude. The PAC-ZNN (16) converges within 3 s and subsequently maintains a convergence accuracy of approximately $10^{-5}$, demonstrating outstanding interference resistance and steady-state performance.

#### 5.1.4. Third-Order Noise Condition

When n=3, as shown in Figure 4d, the differences in convergence and robustness among the six models are further amplified. Except for the proposed model, the other five comparison models exhibit significantly accelerated divergence and demonstrate extreme sensitivity to higher-order noise. Specifically, the NNR-ZNN exhibits a tendency to diverge during the first few seconds. Subsequently, at approximately 4.8 s, the model experiences catastrophic numerical overflow resulting in non-numerical values. This failure is primarily due to the inability of its internal single-integral structure to counteract the explosive accumulation of third-order noise. Furthermore the exponential mapping mechanism inherent in its activation function greatly amplifies uncompensated errors leading to severe gradient explosion and causing the model to fail. The MGRNN similarly suffers from extreme and accelerated divergence issues. In stark contrast, the PAC-ZNN (16) continues to maintain superior performance by converging rapidly and stably. In summary, the PAC-ZNN (16) consistently outperforms all other comparison models in convergence and robustness when solving dynamic linear equations under different noise orders.

### 5.2. Ablation Experiments and Analysis

First, to validate the independent contributions of LMAF (14) and PAC (15), which are two key design elements in PAC-ZNN (16), ablation experiments were conducted in a noise-free environment. A baseline configuration without any enhancement mechanisms served as the reference. As shown in Table 3, when only the PAC term (15) is introduced, ASSRE decreases to $4.464\times 10^{-6}$ and MSSRE drops to $9.161\times 10^{-6}$, indicating that the PAC term (15) significantly reduces steady-state residuals and effectively improves the final solution accuracy. When only LMAF (14) is enabled, the model converges in just 1.6 s while still achieving quantifiable steady-state error levels. This demonstrates LMAF’s (14) significant advantages in convergence speed and dynamic response, enhancing the model’s rapid response capability and convergence efficiency. When both LMAF (14) and the PAC term (15) are enabled, the model converges stably at CT = 1.1 s, achieving ASSRE = $3.471\times 10^{-6}$ and MSSRE = $9.755\times 10^{-6}$. This further demonstrates the complementary benefits of the two mechanisms, enabling the model to achieve superior performance in comprehensive metrics such as accuracy and stable convergence characteristics.

### 5.3. Parameter Sensitivity Experiment

This section focuses on solving the dynamic linear equation A(t)u(t)=b(t), analyzing the optimal state of the model under different parameters through sensitivity experiments.

#### 5.3.1. Sensitivity Experiment of Parameter γ

**Figure 5:**
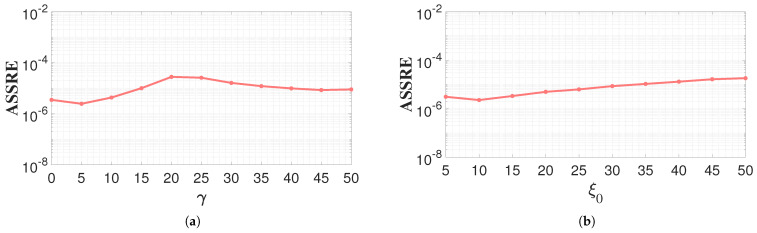
Average steady-state residual error under different values of parameters: (**a**) γ parameter; (**b**) $\xi_{0}$ parameter.

In the experiments, parameters γ were set at equidistant values between 0 and 50, and the average steady-state residual error was calculated. The vertical axis represents the average steady-state residual error, while the horizontal axis denotes different parameter values. As shown in the line chart of Figure 5a, the average steady-state error curve exhibits a minimum value of $2.44\times 10^{-6}$ when γ=5. This result indicates that the PAC-ZNN (16) achieves optimal performance under this parameter setting. Therefore, in subsequent simulation experiments, the gamma parameter of the PAC-ZNN (16) will be set to five.

#### 5.3.2. Sensitivity Experiment of Parameter $\xi_{0}$

The parameter $\xi_{0}$ is a key component of the PAC term coefficient, and its value directly determines system stability. To determine the optimal value, an experimental simulation of a real linear system was conducted. Values of $\xi_{0}$ were taken at equal intervals within the range of 5 to 50, and the average steady-state residual error was calculated. As shown in Figure 5b, the mean steady-state residual exhibits a trend of first decreasing and then increasing with increasing $\xi_{0}$, reaching a global minimum at $\xi_{0}=10$. Initially, a moderate increase in $\xi_{0}$ shifts the system’s poles further toward the left half-plane, thereby enhancing noise suppression and accelerating convergence. However, when $\xi_{0}$ exceeds 10, an excessively large $\xi_{0}$ causes the poles to deviate too far from the imaginary axis, forcing the system into a severely overdamped state and consequently degrading the overall dynamic tracking accuracy. Therefore, in subsequent simulation experiments, the parameter $\xi_{0}$ of the PAC-ZNN (16) will be set at a fixed value of 10.

### 5.4. Dynamic Localization Application Based on AOA Algorithm

To comprehensively demonstrate the advantages of the PAC-ZNN (16) in addressing the two-dimensional dynamic localization task formulated using the AOA strategy, a computer-generated environment is constructed to emulate the corresponding real-world scenario. In this simulated setting, the target node is initialized at u(0)=[2;1], and its theoretical motion trajectory is given as follows:

$$u^{*}\left(t\right)=\left[\begin{array}{c} x^{*}\left(t\right)\\ y^{*}\left(t\right) \end{array}\right]=\left[\begin{array}{c} \left(3+\sin(5t))\cos(t\right)\\ \left(3+\sin(5t))\sin(t\right) \end{array}\right].$$

**Figure 6:**
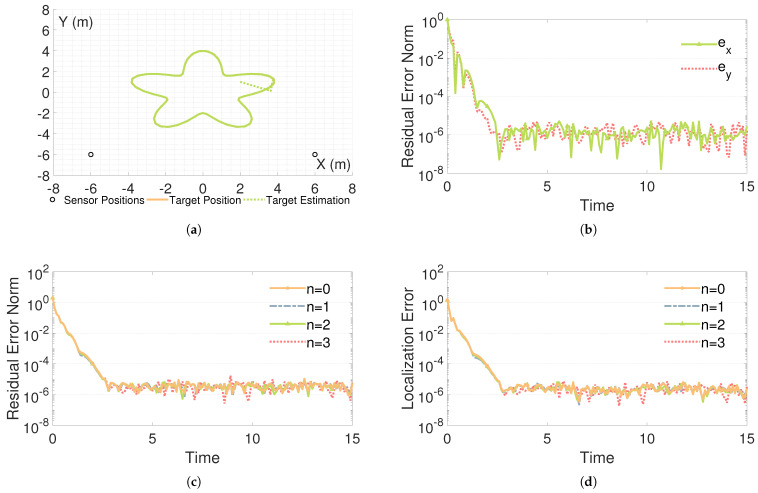
Performance of the PAC-ZNN (16)-based AOA localization algorithm under noise-free and different noise-order scenarios: (**a**) Trajectory overlap comparison; (**b**) Residual error convergence in each direction; (**c**) Residual error under different noise orders; (**d**) Localization error under different noise orders.

As illustrated in Figure 6a, the theoretical motion path is depicted using a solid orange curve, whereas the corresponding estimation produced by the PAC-ZNN (16) is portrayed by the dashed green curve. The high degree of overlap between the two demonstrates that, under a noise-free two-dimensional plane, the computational solution from the PAC-ZNN (16) efficiently and rapidly approximates the theoretical solution. Figure 6b displays the residual error convergence of the PAC-ZNN (16) for solving localization problems in different directions. It is evident that the target node achieves a good approximation of the theoretical trajectory in both the *x* and *y* directions. Specifically, the convergence accuracy in both directions reaches an impressive level of $10^{-6}$. In summary, based on the AOA algorithm, the precision of the PAC-ZNN (16) in two-dimensional dynamic localization tasks has been effectively and rigorously validated.

In the experiment, based on the AOA algorithm, we simulate the noise $\eta \left(t\right)=\sum_{j=0}^{n}a_{j}t^{j}$ of different orders and analyze the change rule of the residual error of the PAC-ZNN (16). In the experiments, the noise order is set from zero to three, and it can be clearly observed from Figure 6c that regardless of the noise order, the convergence accuracy of the PAC-ZNN (16) is excellent, and the residual error can be decreased quickly and stabilized, which fully demonstrates the efficient convergence ability. Even under high-order polynomial noise, the PAC-ZNN (16) exhibits no tendency toward cumulative noise amplification or divergence. This indicates that when solving two-dimensional dynamic localization problems, the PAC-ZNN’s performance remains insensitive to noise order, amplitude, and growth trends. In summary, it can be seen that the PAC-ZNN (16) always has superior noise processing capability and stable residual error control performance in different orders of noise scenarios based on the AOA algorithm, which effectively verifies its applicability and reliability in complex noise environments.

To provide a more intuitive assessment of the actual localization performance of PAC-ZNN (16), Figure 6d shows the absolute localization error of this algorithm under different noise orders. Following the evaluation criteria in [63,64], this metric is defined as the Euclidean distance between the estimated coordinates and the true trajectory, i.e., $E_{AOA}\left(t\right)=\sqrt{{\left(x\left(t\right)-x^{*}\left(t\right)\right)}^{2}+{\left(y\left(t\right)-y^{*}\left(t\right)\right)}^{2}}$. The results show that as the noise order n gradually increases from zero to three, the spatial distance deviation between the estimated position and the theoretical trajectory rapidly decreases within 3 s and eventually stabilizes at an extremely low error level of approximately $10^{-6}$. This fully demonstrates that PAC-ZNN (16) not only minimizes the residual error at the algebraic level but also successfully translates this excellent convergence into highly reliable localization and tracking capabilities in the two-dimensional plane.

### 5.5. Dynamic Localization Application Based on TDOA Algorithm

To clearly demonstrate the superiority of the PAC-ZNN (16) in solving the three-dimensional dynamic localization problem based on the TDOA algorithm, a corresponding simulation scenario is constructed in this study. In this simulated setting, the target node is initialized at u(0)=[1; −1; 2], with its theoretically prescribed motion trajectory defined as

(31) $$u^{*}\left(t\right)=\left[\begin{array}{c} x^{*}\left(t\right)\\ y^{*}\left(t\right)\\ z^{*}\left(t\right) \end{array}\right]=\left[\begin{array}{c} t/2\\ \sin(3t)\\ \cos(3t) \end{array}\right].$$

**Figure 7:**
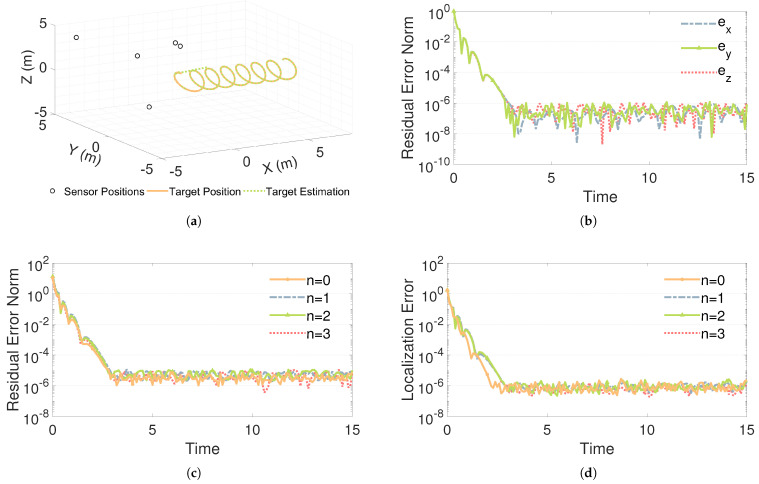
Performance of the PAC-ZNN (16)-based TDOA localization algorithm under noise-free and different noise-order scenarios: (**a**) Trajectory overlap comparison; (**b**) Residual error convergence in each direction; (**c**) Residual error under different noise orders; (**d**) Localization error under different noise orders.

Figure 7a shows the trajectory overlap diagram for solving the localization problem using the PAC-ZNN (16) based on the TDOA algorithm. The orange solid line representing the theoretical trajectory highly overlaps with the green dashed line of the computed trajectory, indicating that the PAC-ZNN (16) achieves high three-dimensional localization accuracy under this algorithm. As shown in Figure 7b, the PAC-ZNN demonstrates outstanding tracking accuracy along the x-axis, y-axis, and z-axis, achieving a convergence precision on the order of $10^{-6}$. The above results therefore fully confirm the accuracy of the PAC-ZNN (16) in performing 3D dynamic localization tasks.

Figure 7c corresponds to the scenarios where the noise order is gradually increased from 0 to 3, respectively. As shown in Figure 7c, the PAC-ZNN (16) exhibits highly consistent response characteristics across zero- to third-order polynomial noise environments, with all curves rapidly decaying to stable values within 3 s. Residual errors under different noise orders fluctuate within a narrow range around $10^{-6}$, with minimal differences in the mean values across curves. Even when increasing the noise order, the model maintains its superior convergence and stability in residual error. This experimental result demonstrates that the PAC-ZNN (16) consistently exhibits outstanding three-dimensional spatial localization capabilities when confronted with noise interference of varying orders.

To further validate the model’s spatial localization capability at the physical level, we define the localization error as the three-dimensional Euclidean distance between the estimated coordinates and the theoretical target trajectory, defined as $E_{TDOA}\left(t\right)=\sqrt{{\left(x\left(t\right)-x^{*}\left(t\right)\right)}^{2}+{\left(y\left(t\right)-y^{*}\left(t\right)\right)}^{2}+{\left(z\left(t\right)-z^{*}\left(t\right)\right)}^{2}}$. As shown in Figure 7d, under constant noise conditions, this localization error converges rapidly within 2.3 s and stabilizes at an accuracy of approximately $10^{-6}$. When subjected to high-order variable-parameter noise interference ranging from first to third order, the actual localization error also decays within about 3 s and maintains the same high-precision level. These experimental results strongly validate that the proposed PAC-ZNN (16) maintains superior and stable three-dimensional localization performance even under complex multi-order noise interference.

### 5.6. Dynamic Localization Application Under Multipath Environments

The preceding sections have validated the positioning performance of PAC-ZNN (16) under adversarial noise in an ideal environment. However, in actual wireless sensor network deployments, the signals received by distributed sensors are highly susceptible to multipath effects [65]. This phenomenon introduces significant measurement errors and degrades the tracking accuracy of the system. To further explore the resilience and robustness of the model in complex environments, this section simulates the positioning and tracking performance of PAC-ZNN (16) under multipath effects that more closely approximate the physical characteristics of real-world scenarios.

#### 5.6.1. Formulation of the Multipath Interference Environment

For 2D positioning based on AOA, multipath effects cause significant random fluctuations in measured angles. The contaminated observation angles can be modeled as

(32) $${\hat{\alpha }}_{i}\left(t\right)=\alpha_{i}\left(t\right)+\varepsilon_{A}\left(t\right),$$

where $\alpha_{i}\left(t\right)$ represents the ideal line-of-sight angle, and $\varepsilon_{A}\left(t\right)$ is the angular dispersion caused by multipath effects.

Similarly, in TDOA-based 3D positioning, signal reflections and scattering due to multipath effects inevitably cause the actual measured distance between a target node and the *i*th anchor sensor node to exceed the true line-of-sight distance. This ranging formula can be formally expressed as

(33) $${\hat{l}}_{i}\left(t\right)=l_{i}\left(t\right)+\varepsilon_{T}\left(t\right),$$

where $l_{i}\left(t\right)$ denotes the ideal line-of-sight distance, and $\varepsilon_{T}\left(t\right)$ represents the forward distance bias introduced by multipath propagation.

#### 5.6.2. Localization Results Under Multipath Environments

For the AOA algorithm, this paper sets the multipath error model as $\varepsilon_{A}\left(t\right)=0.04+N\left(0,0.08^{2}\right)$ radians. The constant term 0.04 represents the baseline angular offset caused by fixed reflectors in the environment, while 0.08 denotes the standard deviation of Gaussian white noise. According to the Gaussian distribution, the total angular error caused by multipath effects is primarily concentrated within the range of [−0.20,0.28] radians. On the other hand, for the TDOA algorithm, the distance multipath interference is modeled as $\varepsilon_{T}\left(t\right)=0.3+U\left(0,1.5\right)$ meters. Here, 0.3 represents the inherent physical delay introduced by the primary reflection path relative to the line-of-sight path in non-line-of-sight propagation, while 1.5 denotes the upper bound of random scattering delay under uniform distribution. This configuration indicates that the overall ranging error caused by multipath effects will exhibit random fluctuations within the range of 0.3 to 1.8 m.

**Figure 8:**
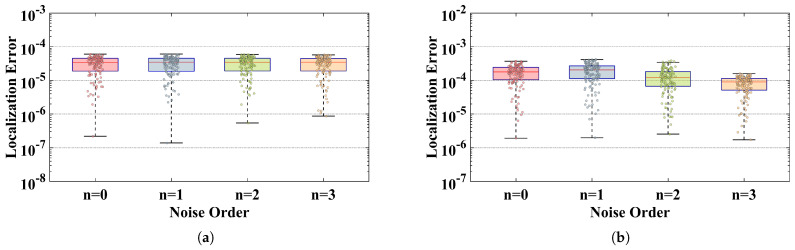
Distribution of positioning errors for PAC-ZNN (16) under different noise levels in multipath environments: (**a**) AOA error distribution; (**b**) TDOA error distribution.

As shown in Figure 8, this paper employs a classic box plot with overlaid scatter points to illustrate the distribution of positioning errors for PAC-ZNN (16) under different orders of noise interference in a multipath environment.

Based on the results in Figure 8a, under the specified angular multipath error model, the median noise positioning error for each order of the two-dimensional AOA algorithm consistently stabilizes near the $3.5\times 10^{-5}$ magnitude. Although the scatter plot exhibits minor error fluctuations caused by random multipath scattering, the box containing the top fifty percent of data remains exceptionally flat and compact. This strongly indicates that the overall error distribution is highly concentrated between the $10^{-5}$ and $10^{-4}$ orders of magnitude. This signifies that PAC-ZNN (16) not only avoids result divergence due to complex multipath effects but also exhibits no error accumulation trend with increasing external noise orders.

Similarly, in three-dimensional positioning scenarios based on the TDOA algorithm, the system faces multipath interference with a wider range of distance offsets and more complex physical delays. Figure 8b clearly shows that the core distribution region of the model error remains stable at around the $10^{-4}$ order of magnitude. Even with increased noise order, neither the upper and lower boundaries of the error distribution nor the box shape exhibit significant deterioration or shift. This fully demonstrates that under multipath interference, the PAC-ZNN (16) positioning performance continues to exhibit exceptional steady-state characteristics and robust resistance against external interference.

## 6. Conclusions

This paper proposes the polynomial anti-noise compensation ZNN (PAC-ZNN) with polynomial anti-noise compensation (PAC) to efficiently address localization problems in wireless sensor networks (WSNs) subject to high-order time-varying noise interference. Traditional models fail to actively learn noise characteristics, leading to increased residual errors as noise intensifies over time. We mitigate the impact of high-order noise by employing PAC term based on the Taylor expansion of the noise function. Additionally, a nonlinear logarithmic mapping activation function is introduced to enhance the robustness and convergence capability of the model. Comparative simulations demonstrate that the PAC-ZNN outperforms other neural dynamic models in suppressing high-order time-varying noise during WSN localization. Consequently, the PAC-ZNN significantly improves the robustness and convergence accuracy of the system, particularly in environments dominated by high-order polynomial noise.

Our method offers significant advantages across various application domains. In urban traffic navigation, the algorithm can robustly track fast-moving targets despite severe multipath reflections caused by buildings. In aerospace exploration, it provides stable trajectory tracking under extreme signal attenuation. For wildlife tracking, its low computational overhead helps extend the battery life of deployed sensor nodes. Despite these advantages, several challenges remain in practical applications. Hardware limitations of IoT nodes and concentrators may degrade system performance. Furthermore, non-ideal transceivers introduce unpredictable phase shifts and variations in signal amplitude. These physical imperfections can distort measurements, sometimes even exceeding the compensation capabilities of the current polynomial.

## 7. Future Work

In future work, we will continue to explore the potential of the PAC-ZNN. On one hand, we will optimize the active learning mechanism for noise features, conduct in-depth research on the dynamic evolution patterns of noise, and improve the adaptive learning strategy for compensation terms to further broaden the range of noise suppression capabilities of the model. On the other hand, we will integrate mathematical principles with the equilibrium characteristics of neural dynamical models to explore the relationship between activation functions and model equilibrium states. This will involve optimizing their structure and parameters to enhance convergence stability.

Regarding the hardware limitations mentioned in the conclusions, we will actively seek algorithmic solutions. By introducing more advanced calibration mechanisms and error modeling methods within the neural dynamics framework, we will strive to compensate for measurement errors caused by non-ideal hardware through algorithmic means. Additionally, we plan to extend the optimized model to a broader range of practical applications to translate our theoretical findings into practical solutions.

## Figures and Tables

**Table 1:** Performance comparison of PAC-ZNN and various neural network models for dynamic localization.

Model	Derivative Driven	Integral Feedback	Adaptive Mapping	Noise Compensation	Robustness Under Noise
LPNN (17)	×	×	×	×	Weak
MZDL1,2 (18)	✓	×	×	×	Weak
FTRZNN (19)	✓	×	×	×	Medium
NIZND (20)	✓	✓	×	✓	Medium
NSRNN (21)	✓	✓	×	✓	Medium
**PAC-ZNN**	✓	✓	✓	✓	**Strong**

**Table 2:** List of main symbols.

Symbol	Description	Symbol	Description
*m*	Number of anchor sensor nodes	$\kappa_{j}\left(t\right),\omega_{j}\left(t\right)$	State variables of the PAC term
*n*	Order of polynomial compensation	$c_{j}$	Coefficients of the PAC term
*r*	Actual noise order	$\gamma ,\xi_{0}$	Design parameters and feedback gains
$a_{j}$	Coefficients of the polynomial noise	$\rho_{j}\left(t\right)$	Intermediate state variable in Laplace transform
*ℏ*	Upper bound of uncompensated noise	$P_{j}\left(s\right)$	Laplace transform of $\rho_{j}\left(t\right)$
V(t)	Lyapunov candidate function	H(s)	Transfer function

**Table 3:** Ablation study of the PAC-ZNN in noise-free environment.

Model Configuration	LMAF	PAC	ASSRE	MSSRE	CT (s)
w/o LMAF and PAC	No	No	$2.059\times 10^{-4}$	$7.974\times 10^{-4}$	1.9
w/o LMAF	No	Yes	$4.464\times 10^{-6}$	$9.161\times 10^{-6}$	2.6
w/o PAC	Yes	No	$1.575\times 10^{-4}$	$1.249\times 10^{-3}$	1.6
PAC-ZNN (16)	Yes	Yes	** $3.471\times 10^{-6}$ **	** $9.755\times 10^{-6}$ **	1.1

## Data Availability

The original contributions presented in this study are included in the article. Further inquiries can be directed to the corresponding author.
